# The prostate cancer detection rates of CEUS-targeted versus MRI-targeted versus systematic TRUS-guided biopsies in biopsy-naïve men: a prospective, comparative clinical trial using the same patients

**DOI:** 10.1186/s12894-017-0213-7

**Published:** 2017-04-05

**Authors:** A. W. Postema, M. J. V. Scheltema, C. K. Mannaerts, R. J. G. Van Sloun, T. Idzenga, M. Mischi, M. R. E. Engelbrecht, J. J. M. C. H. De la Rosette, H. Wijkstra

**Affiliations:** 1 Department of Urology, AMC University Hospital, Amsterdam, The Netherlands; 2 Department of Electrical Engineering, Eindhoven University of Technology, Eindhoven, The Netherlands; 3 Department of Radiology, AMC University Hospital, Amsterdam, The Netherlands

**Keywords:** Prostate cancer, Prostate cancer imaging, mpMRI, CEUS, Contrast enhanced ultrasound, Targeted prostate biopsies

## Abstract

**Background:**

The current standard for Prostate Cancer (PCa) detection in biopsy-naïve men consists of 10–12 systematic biopsies under ultrasound guidance. This approach leads to underdiagnosis and undergrading of significant PCa while insignificant PCa may be overdiagnosed. The recent developments in MRI and Contrast Enhanced Ultrasound (CEUS) imaging have sparked an increasing interest in PCa imaging with the ultimate goal of replacing these “blind” systematic biopsies with reliable imaging-based targeted biopsies.

**Methods/design:**

In this trial, we evaluate and compare the PCa detection rates of multiparametric (mp)MRI-targeted biopsies, CEUS-targeted biopsies and systematic biopsies under ultrasound guidance in the same patients. After informed consent, 299 biopsy-naïve men will undergo mpMRI scanning and CEUS imaging 1 week prior to the prostate biopsy procedure. During the biopsy procedure, a systematic transrectal 12-core biopsy will be performed by one operator blinded for the imaging results and targeted biopsy procedure. Subsequently a maximum of 4 CEUS-targeted biopsies and/or 4 mpMRI-targeted biopsies of predefined locations determined by an expert CEUS reader using quantification techniques and an expert radiologist, respectively, will be taken by a second operator using an MRI-US fusion device. The primary outcome is the detection rate of PCa (all grades) and clinically significant PCa (defined as Gleason score ≥7) compared between the three biopsy protocols.

**Discussion:**

This trial compares the detection rate of (clinically significant) PCa, between both traditional systematic biopsies and targeted biopsies based on predefined regions of interest identified by two promising imaging technologies. It follows published recommendations on study design for the evaluation of imaging guided prostate biopsy techniques, minimizing bias and allowing data pooling. It is the first trial to combine mpMRI imaging and advanced CEUS imaging with quantification.

**Trial registration:**

The Dutch Central Committee on Research Involving Human Subjects registration number NL52851.018.15, registered on 3 Nov 2015. Clinicaltrials.gov database registration number NCT02831920, retrospectively registered on 5 July 2016.

## Background

The incidence of PCa has increased in the 1990’s, due to increased awareness and prolific Prostate Specific Antigen (PSA) screening. Although in the United States this trend has partly reversed, PCa is still the most common cancer in men representing 21% of cancer cases and it is estimated that 180,890 men will be diagnosed with PCa in United States in 2016 [1]. In Europe the incidence has increased, with 417,00 new PCa diagnoses in 2012 [2]. According to current standards, patients with a clinical suspicion of PCa based on elevated serum PSA and/or digital rectal examination should undergo 10–12 systematic transrectal ultrasound (TRUS)-guided prostate biopsies to confirm the diagnosis [3]. These systematic “blind” prostate biopsies lead to a considerable rate of overdiagnosis of clinically insignificant PCa as well as underdiagnosis and undergrading of significant PCa [4]. Image - guided targeted biopsy approaches have been proposed to address these problems [5]. Prostate cancer imaging has thus far developed on two platforms: MRI and Ultrasound. On the MRI platform, multiparametric MRI(mpMRI) in which diffusion - weighted MRI and dynamic contrast- enhanced MRI sequences are added to anatomical T2-weighted imaging has become the standard for PCa imaging [6]. A 2015 systematic review on the accuracy of mpMRI for the detection of prostate cancer found 12 studies and reported a negative predictive value for the detection of clinically significant prostate cancer between 63 and 98% and positive predictive values of 34–68% [7]. However, these results should be interpreted with caution since several used biopsy pathology as the reference standard to calculate sensitivity and specificity. Several meta-analyses have addressed the value of mpMRI - targeted biopsy cores and these show an improved per-core detection rate and a beneficial increased per-patient detection rate in patients with a persistent clinical suspicion after prior negative biopsies [5, 8]. The 2016 update of the EAU (European Association of Urology) guidelines on PCa now recommends performing mpMRI in these patients. However, at this point, the value of using mpMRI and MRI-targeted biopsies at initial biopsy in biopsy-naïve patient is debated [3]. Even though high negative predictive values for detecting significant prostate cancer have been reported, significant disease can still be missed by MRI - targeted biopsy. Furthermore, considerable heterogeneity and risk of selection bias of published results exists. [8, 9]. The EAU guidelines therefore recommend combining systematic and targeted biopsies [3]. The review by Van Hove et al. shows a 1–43% absolute and 2–430% relative increase in per-patient detection rate can be achieved by adding mpMRI - targeted cores to systematic cores [9]. Several methods of using the information obtained by mpMRI to target the region of interest with prostate biopsies exist: in cognitive targeting, the operator makes a visual estimation of where the MRI lesion is located during a TRUS-guided biopsy procedure. Taking in-bore biopsies during MRI scanning reduces the risk of targeting error but due to its magnet time consuming and therefore costly nature, it is not used often. Fusion devices have been developed that register the MRI images and US together, guiding the observer towards the MRI lesion during the TRUS procedure [10, 11].

Prostate cancer may be visible on standard B-mode TRUS. However, the sensitivity is generally reported to be around 11–35% and the positive predictive value is often cited to be between 17 and 57%, although some studies have shown slightly better numbers [12, 13]. Hence, B-mode ultrasound is widely regarded as insufficiently accurate for tumor detection making systematic ultrasound-guided biopsies necessary. A conclusion that is supported by the guidelines [3, 14]. Contrast - Enhanced Ultrasound (CEUS) has been proposed to improve the accuracy of TRUS to detect Prostate Cancer. In CEUS, an intravascular Ultrasound Contrast Agent (UCA) is used to visualize the changes in vascularity that are typical for significant PCa, particularly angiogenesis. It has been demonstrated that angiogenesis is essential for prostate tumors to progress from small indolent lesions below 2 mm in size to clinically significant disease [15]. The UCA’s consist of gas-filled micro bubbles of 1–10 μm with a lipid or protein shell that have an intravascular lifespan of several minutes. A systematic review on CEUS demonstrated a sensitivity and specificity of 70 and 74% or PCa detection. It must be noted that this meta-analysis contains a mixture of biopsy - controlled studies and prostatectomy - controlled studies as well as different variants of CEUS. Of particular interest is the analysis by van Hove et al. that shows a 2–8% absolute or 7–35% relative increase in per-patient detection rate was attained by adding CEUS - targeted cores to systematic biopsy protocols [9]. This analysis indicates improved per-patient detection rates can be achieved with adding CEUS-targeted cores, however CEUS-targeted biopsy at this point cannot replace systematic biopsy. Traditional drawbacks of CEUS are its user-dependency, the limited number of planes that can be visualized in one setting, and the fact that the cues that signify a suspicious focus are subtle and present in the image studies in a matter of seconds. To overcome these drawbacks, computer-aided quantification, a relatively new method to analyze CEUS recordings, is used to assist in the CEUS interpretation [16]. In the present trial, we will use the Contrast Ultrasound Dispersion Imaging (CUDI) method with computer-aided quantification developed at the Eindhoven University of Technology [17]. In short, this method entails constructing per-pixel Time-Intensity-Curves (TICs) during the UCA inflow phase of CEUS recordings. Several parameters calculated from the spatiotemporal distribution of these TICs have shown very promising results in predicting PCa presence with Area Under the Curve (AUC) values reaching up to 0.88–0.89 [17, 18]. These results were obtained by estimating how well CUDI could predict whether pixels belonged to a benign or malignant region of interest using radical prostatectomy specimens as the reference standard. In the present study we will evaluate the value of targeted biopsy procedures with mpMRI and CEUS + CUDI quantitative imaging by performing both procedures with systematic biopsies in the same patients scheduled for initial prostate biopsies. This way we will be able to determine how successful these tools can be used for targeting biopsies and how these targeting procedures compare to each other and to systematic biopsies. Additionally, we will analyze to what extent the imaging tools overlap in the tumors they detect or miss, and therefore to what extent they can be used complementary to each other.

## Methods/design

### Study objectives

#### Primary

- To compare the per-patient (significant) prostate cancer detection rate for mpMRI-US fusion - targeted biopsies and CEUS + CUDI-targeted biopsies with 12-core systematic biopsies.

#### Secondary

- To evaluate the value of using both mpMRI and CEUS + CUDI for targeted prostate biopsies.

### Expected outcomes

Based on previous studies that used a fusion device to take 2–4 mpMRI - targeted biopsy cores in biopsy naïve patients, we expect a per-patient cancer detection rate (CDR) between 40–54% for the mpMRI - targeted biopsies. With the targeted cores, CDRs were achieved that were between 1% absolute higher and 13% absolute lower than the CDRs of systematic biopsies in the same series. Of note is that the biggest series used 3 different methods of targeting the mpMRI cores: cognitive targeting, rigid fusion and elastic fusion. Their best result with targeted cores relative to the systematic cores was achieved with the elastic fusion method (33% CDR for systematic cores and 47% for targeted cores). The Artemis (Eigen, Grass Valley, USA) fusion system that will be used in our trials uses elastic registration. The majority of series have performed a similar analysis in patients with previous negative systematic biopsies or mixed patient cohorts which tend to show a better performance of targeted cores relative to systematic cores [19]. In our biopsy naïve cohort we expect to find a per-patient CDR for all PCa grades that is in the same range as the systematic biopsies. In contrast, we expect the per-patient CDR for clinically significant (Gleason >3 + 4) disease of the mpMRI - targeted biopsies to be relatively higher compared to systematic biopsies.

Limited data is available for the value of CEUS-targeted cores. Van Hove et al. reviewed 6 studies that compared the CDRs for CEUS-targeted biopsies and systematic biopsies [9]. They report that targeted biopsy cores achieved CDRs between 13% absolute lower and 4% absolute higher than systematic cores in these heterogeneous studies with mixed patient groups and various CEUS variants used. None of the studies in this review have included the use of quantitative techniques. A previous study done in our own institution, that retrospectively correlated CEUS imaging with and without quantification with systematic biopsy results, showed improved significant PCa detection with the use of quantification. In 82 patients, 5.6% of biopsy locations that were classified benign on imaging showed clinically significant disease when quantification software was used, compared to 8.5% for CEUS without quantification [20]. Unfortunately, no targeted biopsies were taken in this retrospective analysis. We hypothesize that the use of CUDI quantification software in our current study will result in a CDR for significant disease up to the level of systematic biopsies or better. To our knowledge, there are no published data on the complementarity of mpMRI and CEUS in prostate cancer detection and biopsy targeting. Based on the available data on the separate techniques, we expect that the combination will result in a CDR higher than that of systematic biopsies, especially for the detection of clinically significant disease.

### Study design

This study is a prospective in-vivo study in humans in which we perform MRI imaging and CEUS imaging in biopsy-naïve patients scheduled for prostate biopsies. Targeted prostate biopsies based on these images will be taken besides current standard of care systematic biopsies. Since both imaging modalities and targeted biopsies will be performed in the same patients, every patient effectively serves as his own control. Patients will undergo mpMRI imaging and CEUS imaging approximately 1 week before the scheduled biopsy appointment. During this week, MRI reading will be performed by a specialized uroradiologist with approximately 10 years of prostate mpMRI experience using the European Society of Urogenital Radiology (ESUR) Prostate Imaging Reporting and Data System standardised scoring system, version 2 (PIRADS v2) [21]. The radiologist is blinded to CEUS and CUDI results. The CEUS recordings will be analysed using the CUDI quantification technique. An observer experienced in CEUS of the prostate will read the CEUS recordings and CUDI-maps, while blinded to the MRI results. Before the biopsy procedure, the MRI - based biopsy targets and CEUS + CUDI - based biopsy targets are therefore predetermined independently from each other. During the biopsy session, approximately 1 week after the imaging is recorded, a physician who is blinded to all imaging results and the targeted biopsy procedure will first perform the standard 12-core systematic biopsy to prevent potential bias by post-biopsy haemorrhage seen on ultrasound after targeted biopsies. Then a separate observer, using an MRI-US fusion device, takes a maximum of 4 targeted biopsies from the MRI lesions delineated by the radiologist followed by a maximum of 4 targeted cores from the CEUS + CUDI lesions identified by the CEUS expert. Per-patient CDRs and tumor differentiation grades are compared between each of the biopsy regimens: MRI - targeted biopsies, CEUS + CUDI - targeted biopsies and systematic biopsies.

### Population

Two hundred ninety-nine biopsy-naïve men above the age of 18 years that are scheduled for initial prostate biopsies on the basis of a suspicious DRE and/or elevated serum PSA (above 3 ng/ml) will be included in the study. Patients will be recruited at the AMC University Hospital and all study procedures will be performed at that institution. Exclusion criteria are mostly related to the MRI, the UCA used for CEUS imaging and biopsy procedures(Table 1). Patients will be informed about study procedures, risks and benefits, and are only included after written informed consent has been obtained.

**Table 1:** Inclusion and exclusion criteria

Inclusion Criteria	Exclusion Criteria
1. Age ≥ 18 years 2. Signed informed consent 3. Referred for prostate mpMRI and prostate biopsies	1. Is incapable of understanding the language in which the information for the patient is given 2. Has undergone previous prostate biopsies 3. Active (urinary tract) infection or prostatitis 4. History of any clinically evidence of cardiac right-to-left shunts 5. Receives treatment that includes dobutamine 6. Severe pulmonary hypertension (pulmonary artery pressure >90 mmHg) or uncontrolled systemic hypertension or respiratory distress syndrome 7. Any medical condition or other circumstances which would significantly decrease the chances of obtaining reliable data, achieving study objectives, or completing the study 8. Any (further) contraindication to undergo mpMRI or CEUS imaging

*mpMRI* multiparamteric Magentic Resonance Imaging, *CEUS* Contrast - Enhanced Ultrasound

### Study procedures

#### Multiparametric magnetic resonance imaging

Prior to the mpMRI acquisition, a rectal preparation with a laxative suppository (Bisacodyl) will be performed and just before mpMRI scanning an anti-Peristaltic Drug (Buscopan or Glucagon) will be given. mpMRI will be performed in supine position on a 1.5 Tesla AVANTO® MRI scanner (Siemens Healthcare, Erlangen, Germany) or on a 3 Tesla INGENIA® without endorectal coil (Philips Medical Systems, Best, The Netherlands). The scanning protocol starts with T2-weighted sequences which will be performed in sagittal, coronal and axial planes covering the prostate and seminal vesicles. Then, a single-shot-echo planar diffusion-weighted sequence with fat suppression pulse is acquired and ADC maps are calculated. Finally, dynamic contrast-enhanced images using 0.1 mmol of gadopentetate dimeglumine (Gadolinium DTPA, Gadovist) per kg of body weight, are obtained. More details on the mpMRI conduct can be found in Table 2. mpMRI will be evaluated by a specialized uroradiologist (blinded for CEUS results) on prostate volume and area’s suspicious for PCa. Scoring of suspicion will be performed using PIRADS v2 [21]. All lesions will be marked and delineated for MRI-TRUS fusion using the ProFuse (Eigen, Grass Valley, USA) software package that accompanies the Artemis fusion device.

**Table 2:** MRI conduct

mpMRI Conduct		
Unit	Philips 3 Tesla INGENIA®	Siemens 1.5 Tesla AVANTO®
Slice Thickness		
T2-Weighted	3 mm	3 mm
DW-MRI	4 mm	5 mm
DCE-MRI	2 mm	4 mm
T2-weighted planes	axial, coronal and sagittal	axial, coronal and sagittal
DCE-MRI		
Temporal resolution	5.00 s	3.06 s
Post-processing model	Tofts model using Dynacad (In Vivo, Best, The Netherlands)	Tofts model using Dynacad (In Vivo, Best, The Netherlands)
DW-MRI,		
B-values,	B0, B100, B1000	B50, B800
Imaging sets	ADC, calculated B1500	ADC
Quantitative analysis	primarily qualitative, quantitative in unclear cases	primarily qualitative, quantitative in unclear cases

*mpMRI* multiparamteric Magentic Resonance Imaging, *DW-MRI* Diffusion - Weighted Magnetic Resonance Imaging, *DCE-MRI* Dynamic Contrast- Enhanced Magnetic Resonance Imaging

#### Contrast-enhanced ultrasound and quantification

Contrast Ultrasound scanning will be performed in the left-lateral decubitus position using a Philips IU22 ultrasound scanner with a C10–3V endocavity probe (Philips Healthcare, Bothell, USA) with power modulation at 3.5 MHz and a mechanical index (MI) of 0.06. A total of four CEUS recordings will be made: from the base, mid-base, mid apical and apical planes. The locations of these planes within the prostate are stored using the Artemis fusion device, for future targeting of suspicious lesions. Each of the 2 min recordings will be started following the administration of a 2.4 mL bolus of the contrast agent SonoVue® (Bracco, Geneva, Switzerland) through an intravenous cannula. After each recording a pause of 3 min is observed to allow sufficient UCA breakdown to assess the inflow of the next UCA bolus. The CEUS recordings are anonymized and transferred through a secure connection to the Eindhoven University of Technology for CUDI quantitative analysis. The CUDI maps are transferred back to the physicians to aid in the CEUS interpretation and selection of the biopsy targets. This is done by a CEUS expert, who is blinded to MRI results.

#### Biopsy procedure

Patients are prepared for the biopsy procedure with a 2 day course of fluoroquinolone prophylaxis. The biopsy procedure is performed in the left-lateral decubitus position using the same ultrasound device and probe as used for the CEUS imaging. A first operator blinded to the targeted biopsy procedure planning and imaging results will take a standard 12-core systematic biopsy that includes 2 medial and 4 laterally directed cores of the peripheral zone of the prostate on each side. Then a second physician, using the Artemis fusion device for guidance, takes a maximum of 4 biopsies from targets designated by the radiologist and a maximum of 4 biopsies from the targets designated by the CEUS expert. The needle biopsy cores will be analysed for tumour presence and grading by our institution’s specialized uropathologist in accordance with current pathology guidelines.

### Sample size and statistical analysis

#### Statistical analysis

The per-patient PCa detection rates of both targeted biopsy regimens will be tested separately for non-inferiority against current standard 12-core systematic biopsies using a one sided non-inferiority test for correlated proportions [22, 23]. To evaluate if image- targeted biopsies can be used complementarily, we will also perform the same comparative analysis for the targeted biopsy results combined, compared to the systematic biopsies. Moreover, the per-patient CDR’s of all three biopsy protocols will be directly compared using McNemars test for correlated proportions. The same analyses will be performed including all tumors and including clinically significant tumors only, defined as PCa with a Gleason score ≥3 + 4 = 7.

#### Sample size calculation

The sample size calculation is based on the non-inferiority test between the targeted biopsy procedures and the systematic biopsies. For this test we performed a power analysis, with the detection rate for systematic biopsies set at 45% [24], a power of 80% and the significance level (alpha) of 5%. The maximum allowable difference in detection rate for the targeted biopsies to be considered non-inferior to systematic biopsies was set at 1%. We expect a maximum proportion of discordant pairs of 15% and a detection rate difference of 5%. The required sample size according to these specifications is 260 patients. Based on the estimation that 15% of the patients that are scheduled for biopsies will not receive targeted biopsies due to the absence of PCa suspected lesions on imaging; a total number of 299 patients will be enrolled in the study.

Literature shows that of all positive biopsies about 25% is Gleason >6 [25–27]. Results from our institution differ from literature as only 40% of all the patients with PCa at biopsy have a Gleason score 6 PCa, while 60% of them have a Gleason score >6 PCa. Thus, numbers of positive biopsies and Gleason score > 6 are higher than reported in literature. Based on our historical data we expect that by including 299 patients in the study we will find ~65 cases with a Gleason score = 6 and ~100 cases with a Gleason score > 6. For differentiation between Gleason score we hypothesize that we need at least 40 people in both Gleason grade groups.

### Quality and patient safety

The quality of the data obtained and patient safety will be continuously monitored by the investigators. Periodical (yearly) reporting of study progression and patient safety will be performed to the reviewing Institutional Review Board (IRB). In accordance to section 10, subsection 4, of the Wet Medisch-Wetenschappelijk Onderzoek met Mensen (Medical Research Involving Human Subjects Act), the investigators will suspend the study if there is sufficient ground that continuation of the study will jeopardise subject health or safety. The investigators will notify the accredited IRB without undue delay of a temporary halt including the reason for such an action. The study will be suspended pending a further positive decision by the accredited IRB. The investigator will take care that all subjects are kept informed.

#### Risks and benefits

The mpMRI and TRUS - guided prostate biopsies are already performed routinely in our institution. The extra targeted prostate biopsies are considered to convey minimal additional risk over the systematic biopsy cores that were already planned. When contraindications for mpMRI and necessary preparations for prostate biopsies (proper antibiotic prophylaxis and management of anticoagulant medications) are followed, these investigations are considered safe [3]. After use in thousands of patients, adverse events related to micro bubble UCA’s appear to be transient, mild and rare [28–30]. The most frequent minor side-effects are a transient alteration of taste, local pain at the injection site and facial or general flush (1–5%) [31]. Serious adverse events, which consists of hypersensitivity allergic reactions, are rare (<0.01%) [32]. In a study with SonoVue® in patients with chronic obstructive pulmonary disease, CEUS appeared to be as safe and well tolerated as in a healthy control group [28]. The Committee for Medicinal Products for Human Use has granted SonoVue® a marketing authorization, but it should not be used together with the medicine dobutamine and in pregnant or breastfeeding women (http://www.ema.europa.eu). Although patients are informed about the experimental nature of CEUS imaging, a potential benefit of participation is the real possibility of finding (significant) PCa in one of the targeted biopsies that would otherwise have remained undetected.

## Discussion

Improvement of the PCa diagnostic pathway is necessary, and will likely depend on the advances in PCa imaging. Multiparametric MRI has a recognized value before a second biopsy in case of a persistent clinical suspicion for PCa. The value in biopsy - naïve patients and the possibility to omit systematic biopsies is debated [3]. Contrast -enhanced ultrasound has shown promising results, but should currently be viewed as experimental. This trial will provide data on both these techniques, allowing comparison and assessment of complementarity. The study design chosen is in line with recommendations formulated by the Standards of reporting for MRI-targeted biopsy studies (START) Working Group [33] and by van Hove et al [9]. To validate imaging - targeted prostate biopsy protocols van Hove et al. recommend using either one of two study designs: randomize patients to undergo either systematic biopsies alone or systematic biopsies and targeted biopsies. In the other option every patient undergoes both targeted biopsies and systematic biopsies by separate blinded observers. In our study design all patients undergo both imaging procedures and both targeted and systematic biopsies. The targeted biopsies and systematic biopsies are performed by separate observers. The targeted biopsy locations are predetermined by blinded readers and targeted through a fusion device. To prevent potential bias by post-biopsy haemorrhage seen on ultrasound the targeted biopsies are performed after the systematic biopsies. This way we prevent information gathered from one imaging technique to influence biopsy targeting for the other imaging technique. In both study designs the (significant) PCa detection rates are compared. An advantage of using these study designs is that there is no spectrum bias, i.e. the relevant biopsy population is being studied. Also, there is no observer bias, the investigator reading the images does not already know that there must be a tumor. Both spectrum bias and observer bias occur when translating results obtained from the correlation of imaging and radical prostatectomy specimens towards the primary diagnostic setting. Furthermore, the endpoint in this study design, (significant) PCa detection, is the most relevant endpoint in the clinic. We therefore believe that our trial will provide important and necessary data on one of the most relevant topics in PCa care. This data is gathered in a recommended, standardized fashion to minimize bias and allow data pooling.

## Abbreviations

(mp)MRI: (multiparametric) Magnetic Resonance Imaging
AUC: Area Under the Curve
CDR: Cancer Detection Rate
CEUS: Contrast - Enhanced Ultrasound
CUDI: Contrast Ultrasound Dispersion Imaging
DRE: Digital Rectal Examination
EAU: European Association of Urology
IDM: Interdepartmental Monitoring
IRB: Institutional Review Board
PCa: Prostate Cancer
PSA: Prostate Specific Antigen
TIC: Time-Intensity-Curve
TRUS: Transrectal Ultrasound
UCA: Ultrasound Contrast Agent
